# Supplementary material for: Combinations of Cannabidiol and Δ9-Tetrahydrocannabinol in Reducing Chemotherapeutic Induced Neuropathic Pain
Source: Biomedicines. 2022 Oct 12;10(10):2548. doi: 10.3390/biomedicines10102548 (PMC9599350; doi:10.3390/biomedicines10102548)
Supplement: Supplementary file 1 [file biomedicines-10-02548-s001.zip › biomedicines-1922342-supplementary figures .pdf]

A.

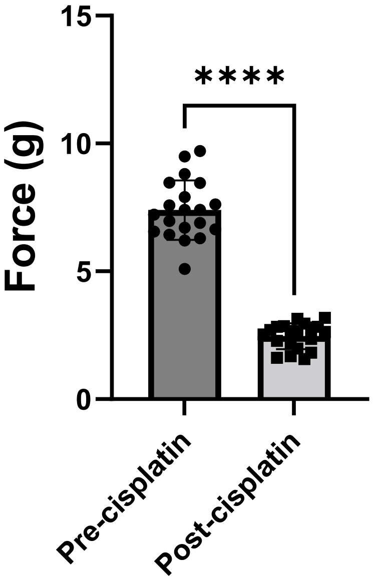

B.

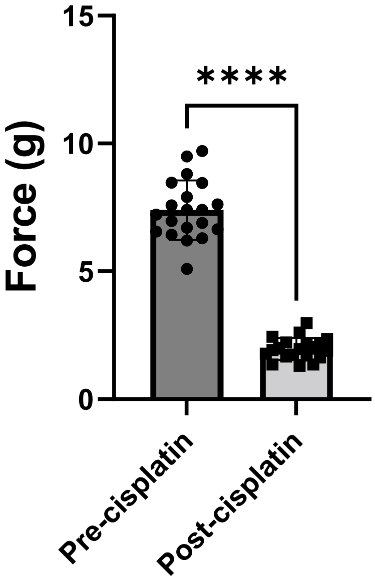

Supplemental Figure S1: von Frey assessment of animals before and after cisplatin treatment. A) Response to von Frey assessment before and after cisplatin for the CBD dose response male mice presented in Figure S1 A. B) As in A except for the THC dose response animals (Figure S1 B). **** p<0.0001 as evaluated by Student's t-test.

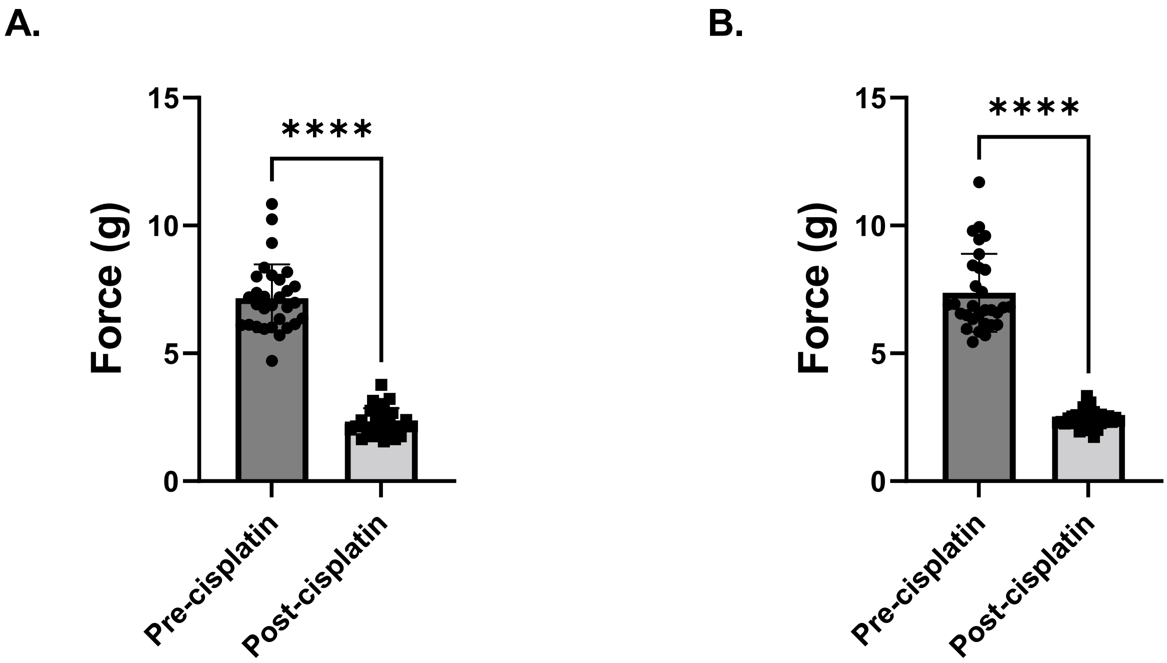

Supplemental Figure S2: von Frey assessment of animals before and after cisplatin treatment. A) Response to von Frey assessment before and after cisplatin for the pure compound treat-ed male mice presented in Figure S2 A. B) As in A except for female mice shown in Figure S2 B. **** $p<0.0001$ as evaluated by Student's t-test.

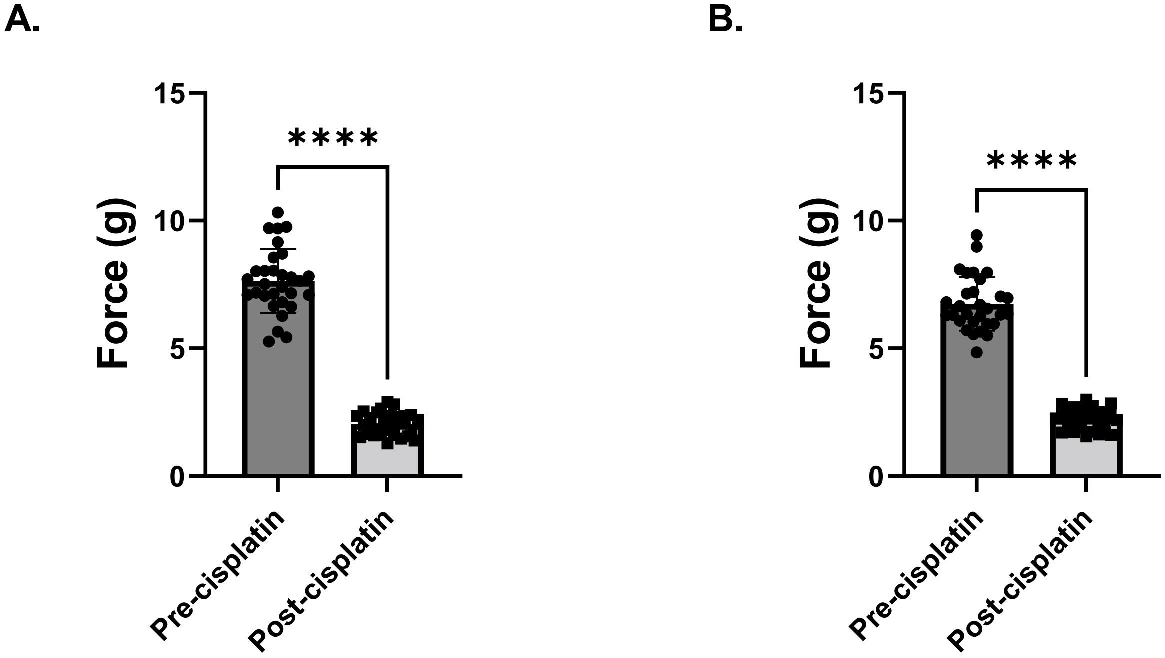

Supplemental Figure S3: von Frey assessment of animals before and after cisplatin treatment. A) Response to von Frey assessment before and after cisplatin for the botanical extract treat-ed male mice presented in Figure S3 A. B) As in A except for female mice shown in Figure S3 B. **** p<0.0001 as evaluated by Student’s t-test.
